# Otoacoustic Emissions in Non-Mammals

**DOI:** 10.3390/audiolres12030027

**Published:** 2022-05-11

**Authors:** Geoffrey A. Manley

**Affiliations:** Department of Neuroscience, Faculty of Medicine, University of Oldenburg, 26129 Oldenburg, Germany; geoffrey.manley@uol.de

**Keywords:** auditory organ, hearing epithelium, basilar papilla, amphibian, reptile, lizard, bird

## Abstract

Otoacoustic emissions (OAE) that were sound-induced, current-induced, or spontaneous have been measured in non-mammalian land vertebrates, including in amphibians, reptiles, and birds. There are no forms of emissions known from mammals that have not also been observed in non-mammals. In each group and species, the emission frequencies clearly lie in the range known to be processed by the hair cells of the respective hearing organs. With some notable exceptions, the patterns underlying the measured spectra, input-output functions, suppression threshold curves, etc., show strong similarities to OAE measured in mammals. These profound similarities are presumably traceable to the fact that emissions are produced by active hair-cell mechanisms that are themselves dependent upon comparable nonlinear cellular processes. The differences observed—for example, in the width of spontaneous emission peaks and delay times in interactions between peaks—should provide insights into how hair-cell activity is coupled within the organ and thus partially routed out into the middle ear.

## 1. Introduction

This review was requested as part of the Special Issue on Otoacoustic Emissions. The intention is to provide those in the field of Audiology with a brief introduction to the facts underlying the presence and the characteristics of otoacoustic emissions in non-mammals to provide part of the framework of understanding mammalian and human emissions. Since the mechanisms underlying the patterns of otoacoustic emissions are still not thoroughly understood and modeled, common characteristics across all groups and specific differences can be used as guidelines in formulating models.

Through recent decades, both the development of powerful CT scanners and a large number of new fossil discoveries, especially from China, have greatly accelerated our understanding of the evolution of land vertebrates. It is now clear that auditory organs, including both middle- and inner-ear structures, have a very complex evolutionary history, with unique features in each major group [1]. Since mammalian middle ears and cochleae are unique [2], and most auditory research has been devoted to their study, they are not the subject of this contribution. Instead, the emphasis is on the living descendants of groups that arose later than did mammalian ancestors and are now commonly known as amphibians, reptiles, and birds. A more accurate systematic organization that parallels their evolutionary history, however, names them Lissamphibia (all modern amphibians), Chelonia (turtles and relatives), Squamata (lizards and snakes), and Archosauria (alligators and their relatives, Aves—birds).

The lineages leading to the modern representatives of these groups have been separated for more than 200 million years and diverged from each other at times when the hearing epithelia of land vertebrate inner ears were small and simple papillae that, in each case, lacked many of the specializations we see in modern representatives [1]. The evolution of specializations, such as elongated hearing papillae and differentiated hair-cell types, thus happened separately in each lineage, and unique combinations of selective pressures induced unique evolutionary trajectories in each group. The differences now observed in inner-ear auditory epithelia necessitate that in this review, each taxon be dealt with separately, which explains the taxonomic arrangement of the details described in the following sections. On the other hand, the middle ears, all of which consist functionally of one ossicle (anatomically with at least two sub-components) [3], will be ignored since there is currently no evidence that middle ears subtly affect the characteristics of otoacoustic emissions (OAE) unless there is an air-pressure difference across the eardrum [4,5].

In spite of the unique group differences in auditory anatomy, it is worth emphasizing at the beginning that the substrate of otoacoustic emission energy lies in the sensory cells—the hair cells—themselves. This explains the universality of otoacoustic emissions (OAE) in all land vertebrates; there are differences between the groups in which types of emissions are more prominent, and spontaneous OAE (SOAE), while measured in all groups, may only occur in one or a few species. Spontaneous otoacoustic emissions are regarded as the most obvious and prominent indicators of the presence of active processes in inner ears [6,7].

OAE of some types, especially the sound-induced DPOAE (distortion-product OAE) and SFOAE (stimulus-frequency OAE), can always be measured, as they are the inevitable result of the nonlinear processing of sound by active hair cells [6]. DPOAE can be easily measure, since their very nature means that the frequency at which they occur can be calculated from knowledge of the frequencies of the two initiating—or “primary”—tones, f_1_ and f_2_, of which f_1_ has the lower frequency. The most prominent and thus most often measured are the cubic distortion products 2f_1_−f_2_ and 2f_2_−f_1_. Even at the most favorable ratios between the frequencies of the primary tones, their sound levels are usually more than 40 dB below the levels of the primaries. SFOAE are also easy to measure since the emission is also induced but by a single tone. The microphone thus measures the tone produced by the loudspeaker plus the emission component at the same frequency. To measure the emission component, it is suppressed by a second tone added very close to the first; the calculated SFOAE emission is the level difference at the emission frequency between the two conditions. The SFOAE technique, being induced by an added tone, also allows phase measurements that can provide additional information on sound processing by the hair-cell system.

The current challenge in understanding the OAE of non-mammalian land vertebrates lies in the differences between these and those measured from mammals and any linkage between inner-ear epithelial structure and the patterns observed in the OAE. This review is only a very brief summary to provide pointers to more information. It will, however, in the final section, indicate where substantial progress has been made, where important commonalities among OAE lie (especially with the OAE of mammals), and in which directions future work may provide interesting new explanatory data.

## 2. The Auditory Epithelia of Non-Mammals

**Frogs**: divided into sub-groups, but of interest here is the fact that the species studied for OAE have two separate auditory epithelia, neither of which has been securely homologized to the single auditory epithelium of all other land vertebrates. The larger frog auditory organ is known as the “amphibian papilla” and on average contains at least a few hundred hair cells. The amphibian papilla shows substantial differences in its size between frog groups. The smaller organ is the “basilar papilla”, which has less than 100 hair cells [8]. Both have tectorial coverings through which the hair-cell bundles are stimulated (review in [9]) and through which presumably the emissions enter the inner-ear fluids.

In **turtles** (Chelonia), the auditory epithelia are among the simplest known. They consist of a more-or-less oval basilar membrane whose length seldom exceeds 1 mm and that is surmounted by a longitudinal strip of hair cells whose stereovillar bundles are all oriented in the same direction and covered by a relatively substantial tectorial membrane. The epithelia in snakes are very similar although it is rather certain that this simple condition in snakes was reached secondarily through simplification of the more complex papillae of their lizard-like ancestors (review in [10]).

**Crocodilians** and **birds** (Archosauria) are closely related, both being survivors of the dinosaur era, but having separated their lineages at an earlier time point. This relationship is evident in the anatomical pattern of the inner ears, which show remarkable and unmistakable similarities [11]. Depending on their body size, the auditory papilla may be more than 5 mm long (more than 10 mm in the highly specialized owls) but is only 1–2 mm in very small birds. It can contain many thousands of sensory hair cells that are arranged in a unique apical-to-basal and neural-to-abneural distribution. The more apical and neural a hair cell lies, the taller it tends to be and the more heavily innervated by auditory nerve fibers (“tall hair cells”). The more basal and abneural a hair cell, the shorter it is and the fewer afferent neural connections it possesses (“short hair cells”). Short hair cells have been defined as those completely lacking afferent neural connections [12]; instead, they have larger efferent connections. These innervation patterns bear a resemblance to the distributions of neuronal types and hair cells seen in mammals, which has led to the suggestion that there are underlying functional reasons that are the same in birds and mammals [13]. While in birds, the shape transition from “tall” to “short” hair cells tends to be gradual, it is more sudden in crocodilians (review in [10]).

In **lizards**, the variety of epithelial forms is large, and although there have been evolutionary convergences (resulting in similar papillae in less-closely related groups), in general, an expert can identify the family or family group to which a lizard species belongs simply by examining its auditory papilla. The largest papillae in modern lizards are more than 2 mm in length and contain > 2000 hair cells (reviewed in [10]). Although it almost certainly arose in deep ancestry from a simple form resembling that of turtles, the ancestral lizard papilla was, in all probability, tripartite. At each end of the original, unidirectionally-oriented hair-cell area, there arose at both ends hair-cell groups in which some were oriented abneurally, while others were oriented 180° reversed—neurally. This resulted in three hair-cell areas that, during the evolution of the various lizard families, have been partially lost, resulting in two areas, or, as in snakes, both “new” areas were lost [14]. While the bidirectional hair-cell bundle condition is the norm in vestibular hair-cell epithelia, in auditory organs, it is only known from lizards. An addition unique feature of lizard papillae is that in the more ancient, normally unidirectionally oriented region, the hair cells only have best frequencies below 1 kHz. The bidirectionally oriented area (if one was lost) or areas, in contrast, respond to the higher frequencies that can extend up to 7 or 8 kHz. Such a separation of frequency ranges is otherwise only known from amphibians and is, again, essentially restricted to below or above 1 kHz. Although the underlying hair-cell physiology is not the subject of this review, it is worth mentioning that the frequency tuning mechanisms in the two areas probably differ. There is clear evidence that in turtles, 1 kHz is the upper frequency limit of all hair cells and is based on electrical tuning [15], and the hair-cell format resembles that of the unidirectional areas in lizards. There is some evidence for electrical tuning in lizard hair cells, but it is currently not clear where it is found and what its upper frequency limit is (e.g., [16,17]). It is of course possible that during the many millions of years of evolution, frequency tuning mechanisms have differentially evolved, which will make the clarification of this concept difficult. In spite of these unknowns, all lizard papillae have retained more than one hair-cell area.

One possible way to classify lizard auditory epithelia (that is, however, not related to the formal classification of the families, suggesting it has underlying functional explanations) is by the shape of the tectorial membrane (TM). In general terms, it can be said that the TM can be continuous and substantial and is so in many, but by no means all, larger papillae (teiids, varanids) and rarely so in smaller papillae. Some large papillae (geckos, skinks) and many smaller ones (various families) cover their higher-frequency, bidirectional hair-cell areas with a chain-of-beads kind of TM, each “bead” being known as a sallet; each is connected by a thinner strand to each neighbor. Many very small papillae (agamids, iguanids, anguids) have abandoned the TM altogether in these hair-cell areas, and what remains is a small TM unit only covering the unidirectional, low-frequency area. These have become known as “free-standing hair-cell” areas. The type of TM can even vary within one family. These patterns in lizard anatomy are explained here in detail because there is a correlation between the TM form and the patterns of SOAE frequency spectra [18].

## 3. Otoacoustic Emissions in Non-Mammalian Land Vertebrate Groups

### 3.1. OAE of Amphibians

**Figure 1:**
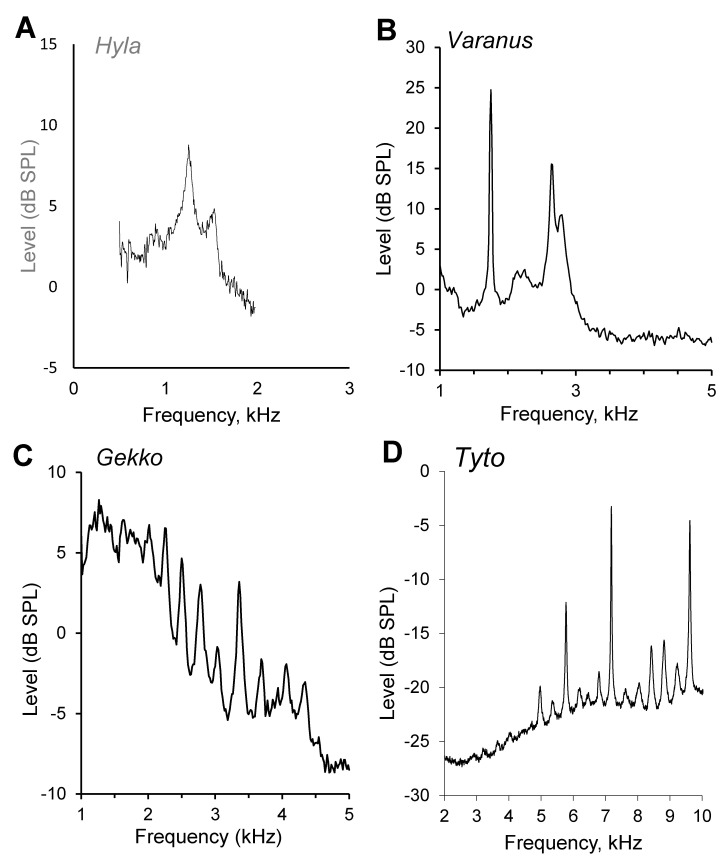
Averaged spectra of spontaneous otoacoustic emissions in (**A**) a frog, *Hyla cinerea* [19]; (**B**) the monitor lizard, *Varanus exanthematicus* (with a continuous tectorial membrane) [23]; (**C**) the gecko *Gekko gecko* (with tectorial sallets) [24]; (**D**) the barn owl *Tyto alba* [25,26]. Note the differences in scaling of the frequency and level axes.

Modern amphibians form several sub-groups, but OAE have only been investigated in frogs. Earlier neurophysiological work had identified the amphibian papilla of frogs as the origin of low- and mid-frequency responses (species-specific but generally below about 1 kHz), with the basilar papilla as the origin of high-frequency responses often exceeding 2 or 3 kHz [19]. While DPOAE have been measured from both auditory papillae, their amplitudes were highest for the upper portion of the frequency range of the amphibian papilla (where SOAE also originate, Figure 1) and for the frequencies processed by the basilar papilla [19]. Low-level DPOAE in frogs are physiologically vulnerable [20]. No DPOAE were measurable in the frequency range that is known from neurophysiological data to be above that of the amphibian papilla but below that of the basilar papilla, in other words, outside the ranges of both papillae. As also found in neural data, the preferred response range of the basilar papilla was much narrower than that of the amphibian papilla. Thus, DPOAE offer a non-invasive objective technique that reveal some of the processing characteristics of the auditory papillae [21].

### 3.2. OAE of Turtles and Their Relatives

Unpublished thesis data [22] examined Pseudemis (Trachemys) scripta, the red-eared turtle, for SOAE and DPOAE and found neither to be above the (low) noise level of the system. While the absence of SOAE was not surprising (no SOAE have been found in lizards below 1 kHz, which is the upper limit of hearing in turtles), that of DPOAE was. Hearing thresholds of this species are, however, high (above 40 dB SPL: review in [10]). The middle-ear system of Chelonia is also rather insensitive, and given that sound levels inducing DPOAE must traverse the middle ear twice, and the highest primary-tone level used was 65 dB SPL, it is very likely that any DPOAE were simply too weak to exceed the noise levels.

### 3.3. OAE in Birds and Crocodilians

The first evidence of the existence of active processes in non-mammals were reports of preferred intervals in the spontaneous activity of auditory-nerve fibers [27] and of swept-tone OAE in the starling (one kind of synchronously-evoked emission or SEOAE; [28]). Swept-tone OAE are measured using a series of slow frequency sweeps of decreasing sound pressure. The levels obtained at different frequencies are then mathematically predicted from those at the highest-level sweep, under the assumption that at the highest levels, the relative pressure of the emissions is negligible. Thus, the lower the swept-tone level, the more prominent are any divergences from the expected levels. The SEOAE measured were most prominent at higher frequencies and were suppressible by added tones, as previously shown in the crocodilian *Caiman* [29]. In the starling, the suppression tuning curves showed a very sharp frequency selectivity similar to that seen in the excitatory tuning of single primary auditory neurons in the same species [30]. The latter clearly suggested that the emissions originated from local groups of active hair cells.

These early indications of active processes in archosaurians were supplemented by more comprehensive data on DPOAE of birds, specifically in the starling and chicken [31]. DPOAE in the birds showed many of the characteristics already measured in mammals, such as the complexity of their dependence on the frequency ratios and levels of the primary tones. These complexities have, in mammalian data, been explained through the phase interference between more than one emission source, but these phase complexities have not yet been examined in non-mammalian data.

The levels of the DPOAE were usually highest at intermediate frequency ratios (roughly 1.2 to 1.4), and the slopes of the output function always lay below the increase in the primary levels. As shown earlier in starlings, the DPOAE were suppressed by added third tones in a frequency-selective fashion, mostly with the best by frequencies near f_1_. This is different from the DPOAE of mammals, which are generally best suppressed by frequencies near f_2_ [32]. In addition, unusually, compared to mammals, the DPOAE were highly sensitive to the level of anesthesia, necessitating measurements in awake animals. The compound action potential of the auditory nerve was even more sensitive to the anesthesia level [31].

DPOAE 2f_1_−f_2_ was also measured in the barn owl using f_1_ frequencies up to 9 kHz, near the species’ upper frequency limit of hearing [33]. In some cases, the DPOAE levels were only 37 dB below those of the primary tones. The optimal primary-tone frequency ratios varied strongly among the different frequency regions investigated, with the largest optimal ratios in the mid-frequencies near 6 kHz. DPOAE levels were suppressed in a frequency-selective way by third tones, with the best suppressive frequencies near f1. The frequency selectivity of the suppression manifest itself through V-shaped tuning curves with Q_10dB_ values that increased with the frequency of the suppressed DPOAE, up to nearly 16. This selectivity strongly resembles the selectivity of auditory-nerve fiber excitation in this species [34].

In none of the experiments with archosaurs were SOAE measurable, with one dramatic exception—a hearing specialist, the barn owl. The barn owl showed very prominent patterns of SOAE that reflect its exceptional hearing range and sensitivity and its highly specialized cochlea, 50% of which is taken up by an expanded high-frequency region that is referred to as an auditory fovea [25,26,35]. In the 1997 study [25], peak sound-pressure levels measured were up to 10.3 dB SPL, and their center frequencies lay between 2.3 and 10.5 kHz (Figure 1D). In the later studies that had a lower noise level, larger numbers of SOAE peaks became evident. SOAEs were found in 100% of ears, with an average of 12.7 SOAEs per ear. Remarkably, across the whole SOAE frequency range of 3.4 to 10.2 kHz, the distances between neighboring SOAEs were relatively uniform, with a median distance of 430 Hz. The majority (87.6%) of SOAEs were recorded at frequencies between 5 and 10 kHz and thus derived from a specialized area previously described as constituting an auditory fovea [36]. Most of the SOAE, even large peaks, showed statistical characteristics of filtered noise rather than of oscillators. SOAE frequencies were temperature sensitive; the frequency shifted up with a rise in temperature by an average of 0.039 oct/°C.

External tones suppressed the SOAE, an effect that, as in the suppression of DPOAE of this species and other birds, was highly frequency selective. Details of the suppression tuning of SOAE in barn owls were studied by Engler et al. [35], who quantified suppression effects by deriving suppression tuning curves with a criterion of 2 dB suppression. Interestingly, smaller SOAEs tended to require a higher sound level to be suppressed. The tuning was V-shaped and sharp. Between 5 and 10 kHz, the median tuning Q_10dB_ value (a standard measure of tuning selectivity calculated as the most sensitive frequency divided by the bandwidth 10 dB above the best threshold) was 4.87 and thus lower than that of owl single-unit neural data. The frequency-threshold curves of auditory-nerve fibers and the suppression of SOAEs thus differed in some respects in their tuning characteristics, indicating that SOAE suppression tuning in the barn owl may not fully reflect neural tuning in primary auditory nerve fibers.

SOAE and DPOAE were both influenced by tones to the contralateral ear, indicating a frequency-selective influence of efferent auditory nerve fibers on the hair-cell activity [37]. The effects were complex, however, corresponding to different patterns of activation observed in efferent neurons of the avian brainstem [38].

SFOAE were also measured in the barn owl [26] and showed an interesting pattern with regard to the SOAE peaks in the spectrum in the ear canal. Between adjacent SOAE peak frequencies, the phase accumulation of the SFOAE clustered around an integral number of cycles. This had previously been demonstrated in data from human ears [39]. A comparison between SFOAE phase-gradient delays (near 2 ms), the typical distance between SOAE peaks of 430 Hz, and auditory nerve fiber frequency selectivity suggested that in the barn owl, the OAE delay can be attributed to the frequency tuning of the auditory epithelium.

### 3.4. OAE in Lizards

The first measurements of OAE in lizards were made by [40] in the alligator lizard. Unfortunately, the noise level in their system made systematic measurements of the DPOAE difficult, especially as there was a very dominant component of 2f_1_−f_2_ that was insensitive to acoustic overstimulation and thus likely not of hair-cell origin. Nonetheless, it was apparent when comparing the spectra produced in the intact animal to those after “basilar membrane destruction” that substantial cubic distortion products were generated in the healthy system.

**Figure 2:**
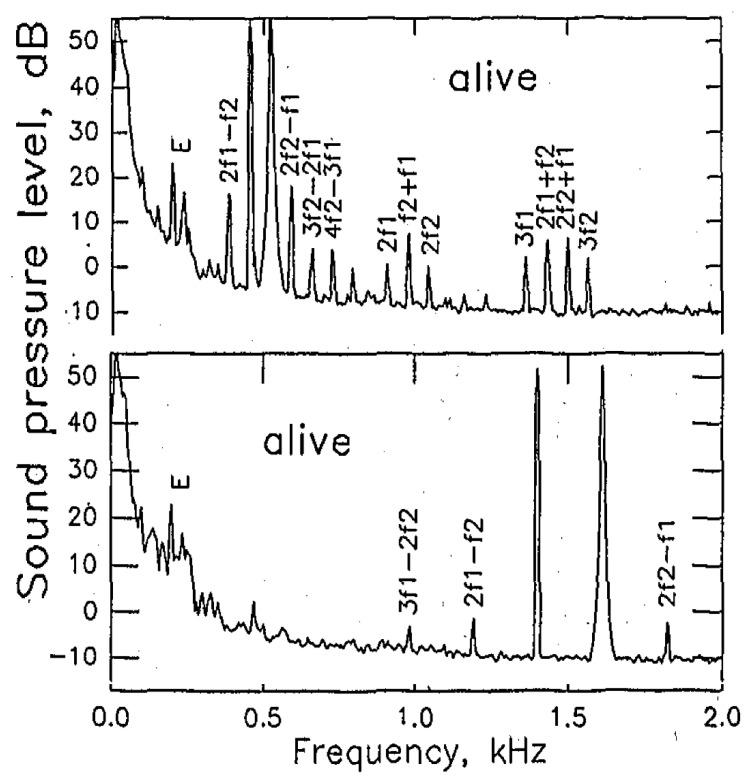
DPOAE spectra produced by two-tone stimulation in the skink *Tiliqua rugosa*. Top panel: low-frequency stimulation with f_1_ and f_2_ near 0.5 kHz. Lower panel: higher-frequency stimulation, with f_1_ and f_2_ near 1.5 kHz. E, electrical noise. Partially after [41].

**Figure 3:**
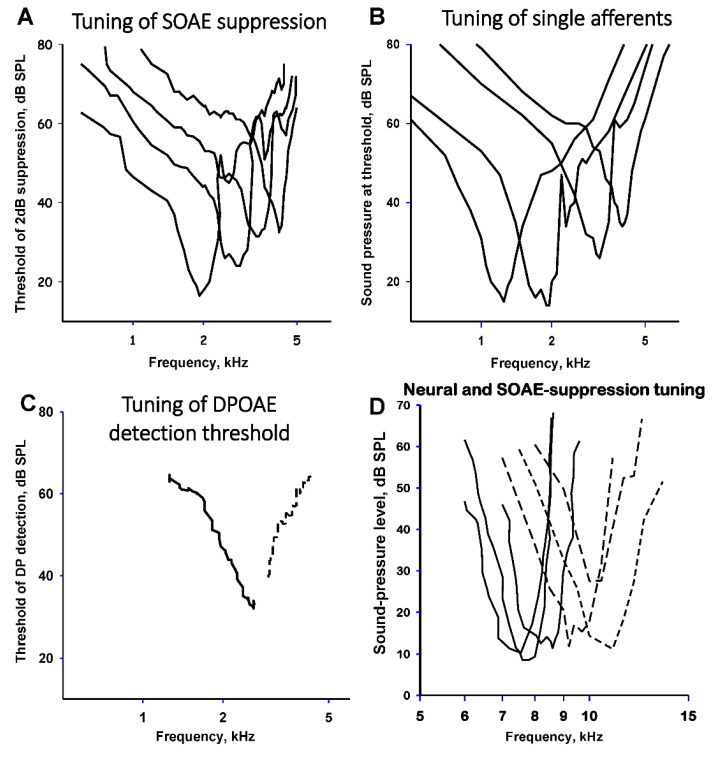
A comparison of different measures of frequency selectivity tuning in (**A**) the suppression of four SOAE, (**B**) threshold tuning of four single auditory-nerve fibers, (**C**) DPOAE detection thresholds for 2f1−f2 (continuous line) and 2f2−f1 (dashed line) in the Bobtail skink *Tiliqua rugosa,* and (**D**) three single auditory nerve fiber tuning curves (continuous lines) and suppression of three SOAE peaks (dashed lines) in the barn owl *Tyto alba*.

An extensive study was made in a large skink, *Tiliqua rugosa* [41,42] (Figure 2 and Figure 3). This species has a 2 mm-long auditory papilla that contains about 2000 hair cells. A small proportion of those are in an area that responds to frequencies below 1 kHz, the majority extend along the main area responding to higher frequencies in a tonotopic manner, up to about 5 kHz (temperature dependent). The low-frequency area is covered by a single, large mass of tectorial material, whereas the longer high-frequency area is covered by a chain of sallets. Each sallet is only attached to maximally two rows of hair cells arranged across the papilla and thus links hair cells that have almost identical best frequencies. The spectra produced in the ear canal by single- and two-tone stimulation differed depending on whether the low- or the high-frequency areas were primarily stimulated (Figure 2). The low-frequency region produced more complex tonal mixtures, and only some of those components disappeared after the death of the animal.

The lowest levels of the primary tones needed to reach threshold levels of DPOAE (two standard deviations larger than the noise at that frequency) closely resembled the tonal levels needed to reach threshold in auditory nerve fibers of this species, suggesting that the tones in each case just begin to stimulate hair cells, suggesting these as the origin of the responses (Figure 3B,C) [41]. With respect to their complexity, the level-response curves closely resembled those of mammals and likewise indicate that they result from the interaction of more than one component in the generation of the final DPOAE. The suppression of distortion products by third tones indicated frequency selectivity that bore a remarkable resemblance to the excitatory tuning curves of single auditory-nerve fibers [42,43], as indeed did the thresholds necessary to generate the distortion products. Interestingly, the levels of DPOAE were not only sensitive to hypoxia, indicating their physiological origin, but were also influenced by the presence or absence of SOAE at the individual frequencies (see below for SOAE).

For the last 29 years, SOAE have been extensively studied in a range of lizard species [4,5,9,14,18,23,24,44,45,46,47,48,49,50,51,52,53,54,55,56,57,58,59,60,61,62]. In this brief review, only some main features of these studies can be covered.

First, SOAE were found in all species and in almost all ears. This sets lizards apart from other amniote groups as being by far the most robust generators of SOAE. In all species in which comparisons can be made, the frequency range and tuning properties of single auditory-nerve fibers resembled those of SOAE and their suppression in the same species. Another common feature was SOAE level facilitation (up to more than 10 dB) through additional tones, generally at frequencies outside of the frequency-level areas that produced suppression. Facilitation of this prominent kind has not yet been reported from mammalian or avian data.

Across all species, the number of SOAE spectral peaks per ear varied from 2 to 15, and their center frequencies lay between 0.75 and 7.7 kHz (Figure 1B,C). Importantly, the number of peaks roughly correlated with the structure of the hearing organ. In those species with an unspecialized tectorial membrane covering all hair cells (such as in teiids and varanids), only between two and seven SOAE peaks were found per ear (Figure 1B). These peaks were, however, generally of high sound levels, up to 27 dB SPL. Species that lack a continuous tectorial membrane and instead have tectorial sallets or no tectorium at all across most of the papilla tend to have a larger number of peaks that are of smaller amplitude, maximally up to 10 dB SPL (Figure 1C). This comparison across a broad range of species and anatomies suggests that the tectorial membrane is a key element in linking hair cells with respect to their frequency tuning [63]. Continuous tectorial membranes would be able to link hair cells across a range of preferred frequencies, whereas sallets link only hair cells with close preferred frequencies. Hair cells that have no tectorium would only be linked by the surrounding fluids.

SOAE peaks can be generated by very small numbers of hair cells. In the green anole, for example, in whom the tiny auditory papilla of which has a frequency range up to more than 7 kHz and a total of only about 140 hair cells, evidence suggests that some small peaks are attributable to only two or 3 = three hair cells [52]. In lizard papillae that lack a tectorial membrane, such SOAE peaks are generated by temporary groups of hair-cell “alliances”, and these change over time, resulting in unstable spectral peaks [57].

**Figure 4:**
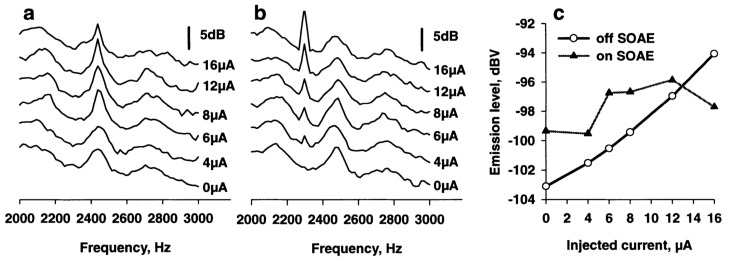
The effects of injected AC current at (**a**) the frequency of an SOAE peak or (**b**) at a frequency with no SOAE peak. The traces in a and b are shifted to avoid overlap. In (**c**) are shown the amplitudes reached in both cases as a function of the injected current. From [54].

In the three species for which frequency maps of the hearing organ are known (the Bobtail skink, the Texas alligator lizard, and the Tokay gecko, the range of SOAE peak frequencies covers the “high-frequency” hair-cell area of the auditory papilla. The hair cells in these areas are generally arranged with their bundles oriented 180° opposed to each other, with some in an orderly fashion and others more unevenly. It is likely that the active process in the bundles can induce a mechanical response in the oppositely-oriented hair cells in a self-sustaining and self-regulating process that continuously generates very low-level sounds that are transmitted through the middle ear and appear as (spontaneous) peaks in sound spectra of the ear canal. That the hair cells in such local areas are active is suggested by experiments using the electrical stimulation of hair cells [54]: AC current injected at frequencies at which there were SOAE produced smaller increases in sound level at that frequency than current injected at frequencies between SOAE peaks (Figure 4).

Studies of isolated lizard papillae indicated that acoustic stimuli activate hair cells through a side-to-side movement of the auditory papilla and, if present, its tectorial structure [64,65]. Self-induced activity of the hair-cell bundles through an active process would presumably also result in such movements. Experiments using complex current injection patterns and simultaneous low-frequency stimulation to selectively bias the hair-cell groups with opposing bundle orientations during each half-cycle of the sound indicated that without sound, only very small electrically induced OAE can be generated. This is because under these conditions, the opposing mechanical energy generated by the two groups mostly cancels out. With sound biasing, however, the OAE generated during each half cycle become much larger (they are out-of-phase) through the release from cancellation. This is strong evidence that the active processes producing OAE in lizards resides in the bundles [54].

Compared to the SOAE of humans (where SOAE frequency bandwidths are ~1 Hz), lizard SOAE have very wide bandwidths; their frequency varies rapidly over a wide range but spends more time near the center of that range. This poor frequency stability is accompanied by a large effect induced by changing the body temperature, an effect also seen in avian SOAE. In addition, under the influence of external tones, lizard SOAE may shift their center frequency up to several hundred Hertz, again indicating their unstable frequencies. Interactions can occur between SOAE peaks, and these have sub-millisecond latencies [50], indicating the existence of a fast and unstable feedback loop. In humans, these interactions also occur but on a ten-times larger time scale, resulting in highly stable and thus narrow SOAE peaks.

## 4. Common Factors Underlying OAE

The available data on otoacoustic emissions of mammals and non-mammals indicate the presence of strong commonalities underlying generator mechanism of OAEs across all vertebrates despite the absence in non-mammals of morphological and functional features (e.g., a traveling wave) that are thought to be essential to mammalian cochlear mechanics.

The main differences between OAE of mammals and non-mammals are:

1. The main location of tonal suppressive effects (by frequencies near f_1_ in non-mammals, but near f_2_ in mammals);
2. Data on the underlying statistics are inconclusive with regard to whether they indicate that the generators are oscillators (mammals, some non-mammalian data) or not (some other non-mammalian data, including some from barn owls). This is complicated by the influence of widely varying peak amplitudes on the analysis;
3. Interactions between peaks are much faster in lizards than in mammals. This is responsible for at least some of the spectral broadness of SOAE peaks in lizards, their temperature sensitivity, and the changes of frequency due to suppression/entrainment by added tones.

Despite these and other differences, which still require explanations, OAE indicate the details of sensitivity and frequency selectivity of the local area of the cochleae in both mammals and non-mammals and are thus “*good and sensitive indicators of cochlear integrity*” [41]. Although some models of lizard basilar papillae that carry salletal tectorial membranes have been successful in explaining SOAE spectral patterns [64,65,66,67], there is obviously a need for more comprehensive modeling studies of the patterns of all OAE from non-mammalian papillae. One of the most promising approaches would be the expansion of comparative studies across species and vertebrate groups [26,68] and the expanded use of mice mutants that, for example, show variation in the structure of the tectorial membrane [69].

## Data Availability

Not applicable.
